# Millisecond Timescale Motions Connect Amino Acid Interaction Networks in Alpha Tryptophan Synthase

**DOI:** 10.3389/fmolb.2018.00092

**Published:** 2018-11-08

**Authors:** Kathleen F. O'Rourke, Jennifer M. Axe, Rebecca N. D'Amico, Debashish Sahu, David D. Boehr

**Affiliations:** Department of Chemistry, The Pennsylvania State University, University Park, PA, United States

**Keywords:** allostery, amino acid networks, enzyme dynamics, enzyme regulation, protein NMR, relaxation dispersion, tryptophan synthase

## Abstract

Tryptophan synthase is a model system for understanding allosteric regulation within enzyme complexes. Amino acid interaction networks were previously delineated in the isolated alpha subunit (αTS) in the absence of the beta subunit (βTS). The amino acid interaction networks were different between the ligand-free enzyme and the enzyme actively catalyzing turnover. Previous X-ray crystallography studies indicated only minor localized changes when ligands bind αTS, and so, structural changes alone could not explain the changes to the amino acid interaction networks. We hypothesized that the network changes could instead be related to changes in conformational dynamics. As such, we conducted nuclear magnetic resonance relaxation studies on different substrate- and products-bound complexes of αTS. Specifically, we collected ^15^N R_2_ relaxation dispersion data that reports on microsecond-to-millisecond timescale motion of backbone amide groups. These experiments indicated that there are conformational exchange events throughout αTS. Substrate and product binding change specific motional pathways throughout the enzyme, and these pathways connect the previously identified network residues. These pathways reach the αTS/βTS binding interface, suggesting that the identified dynamic networks may also be important for communication with the βTS subunit.

## Introduction

Allosteric regulation is a common means of adapting to changing environmental conditions and cellular requirements (Nussinov et al., 2013). In classic views of allostery (Monod et al., 1965; Koshland et al., 1966), effector binding at a distal site changes protein structure to affect function. For enzymes, these structural changes can affect substrate binding, catalytic efficiency and interactions with other macromolecules. In the “mechanical linkage” model, signals from allosteric effectors propagate through sequential structural changes from the distal site to other regions of the protein (Yu and Koshland, 2001). Amino acid residues that are key to these structural transitions comprise an amino acid interaction network (Süel et al., 2003; Amitai et al., 2004; Böde et al., 2007; Grewal and Roy, 2015; Dokholyan, 2016; O'Rourke et al., 2016). This well-ordered view of progressive structural changes appears to stand in contrast to those systems in which allosteric ligands change protein function but do not substantially change protein structure (Popovych et al., 2006; Das et al., 2009; Tzeng and Kalodimos, 2009; Capdevila et al., 2017; Saavedra et al., 2018). In these systems, allosteric effectors may change structural dynamics or conformational sampling to affect interactions with other molecules. In this “dynamically driven allostery” (Cooper and Dryden, 1984; Reinhart et al., 1989; Petit et al., 2009; Motlagh et al., 2014; Kornev and Taylor, 2015; Nussinov and Tsai, 2015; Tzeng and Kalodimos, 2015; Guo and Zhou, 2016; Saavedra et al., 2018), key residues may still guide changes to protein structural dynamics (Rodgers et al., 2013; McLeish et al., 2015; Capdevila et al., 2017).

**Figure 1:**
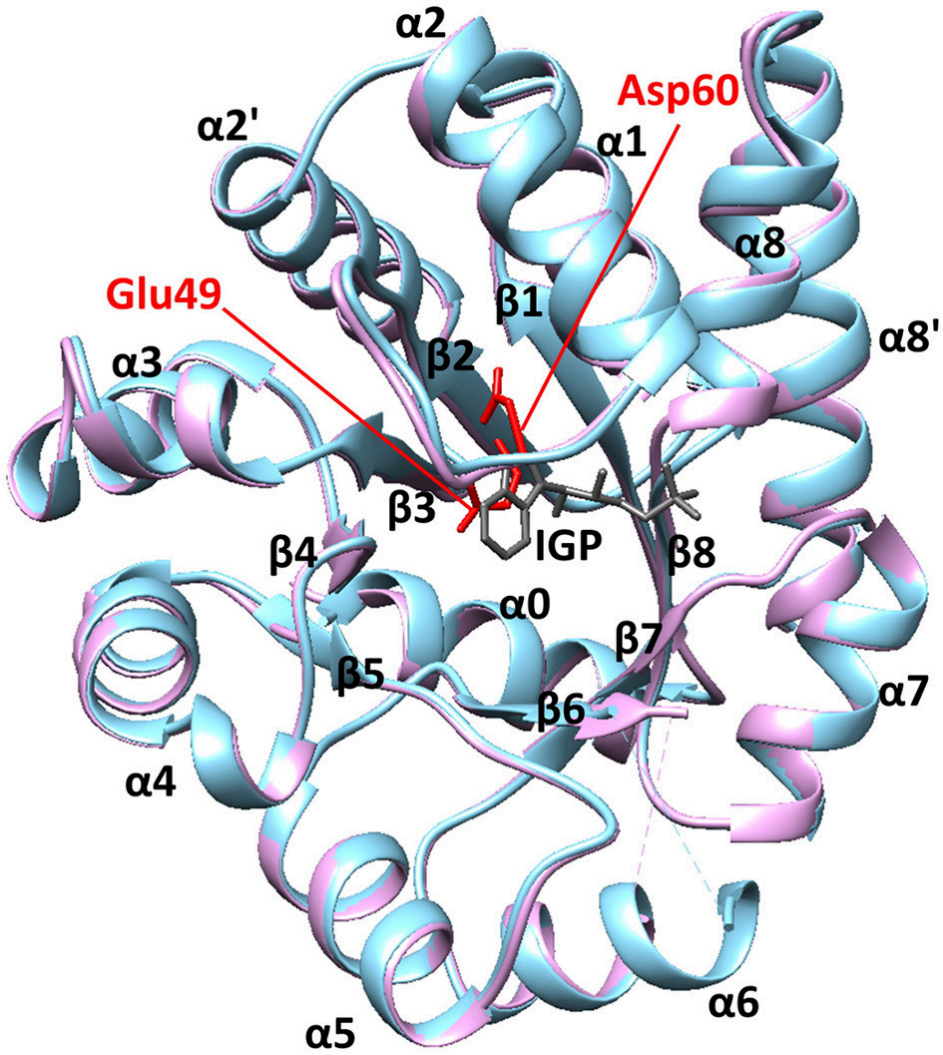
The structure of αTS. These X-ray crystal structures are from *Salmonella typhimurium*, which has 85% sequence identity with *E.coli* αTS used in the NMR experiments. The blue and pink structures represent the ligand-free (PDB 1KFJ) and IGP-bound (PDB 2RHG) states, respectively. Binding of IGP substrate does not substantially change the ground-state αTS structure in the αTS-βTS tryptophan synthase complex.

We have previously used the alpha subunit of tryptophan synthase as a model system for understanding amino acid interaction networks in proteins (Axe et al., 2014). Tryptophan synthase is composed of both alpha (αTS) and beta (βTS) subunits aligned in an α*ββα* manner (i.e., a heterotetramer ~143 kDa in size) and is known for the ability to channel the αTS product indole directly into the active site of βTS through a 25 Å hydrophobic tunnel (Dunn, 2012). The αTS enzyme catalyzes the retro-aldol cleavage of the C3′-C3 bond of indole-3-glycerol phosphate (IGP) to form indole and glyceraldehyde-3-phosphate. The αTS enzyme has a (β/α)_8_ or TIM (triose phosphate isomerase) barrel structure with three additional α-helices (Figure 1). The α0 helix is located at the N-terminus of the protein, the α2′ helix (residues 62–74) is located between the β2 strand and the α2 helix, and the α8′ helix is located between the β8 strand and the α8 helix. Like other TIM barrel enzymes (Sterner and Höcker, 2005), the active site consists mostly of residues on the inner β-strands and the βα loops that connect β-strands to the following α-helices. The αβ loops that connect the α-helices to the β-strands tend to be shorter, and important for structural stability (Sterner and Höcker, 2005). Major structural changes in αTS are localized to the β2α2 and β6α6 loops. In the absence of βTS, the β2α2 loop extends through the α2' helix to include residues 52–77 (Nishio et al., 2005). The β6α6 loop encompasses residues 179–192 and is disordered in the absence of ligands (Kulik et al., 2002; Ngo et al., 2007; Barends et al., 2008; Lai et al., 2011). Upon binding IGP, αTS forms a closed conformation in which these loops make important hydrogen bonding interactions between Ala59, Asp60, and Gly61 on the β2α2 loop and Thr183 on the β6α6 loop. These events are likely important for positioning the catalytic residues Glu49 (on the β2 strand) and Asp60 (Figure 1).

We previously used NMR chemical shift covariance analysis (CHESCA; Selvaratnam et al., 2011; Boulton et al., 2018) to identify amino acid interaction networks in αTS, in the absence of βTS (Axe et al., 2014). Perturbation of these networks was shown to modulate αTS catalytic activity (Axe et al., 2014) and functional interactions with βTS (data not shown). Intriguingly, the amino acid interaction networks were substantially different between the apo *resting* state and the *working* state enzyme, which represents a catalytically-active state under a 4:1 dynamic chemical equilibrium between substrate- and products-bound enzyme (Axe and Boehr, 2013). The lack of structural differences between ligand-free and IGP-bound αTS suggested that changes in αTS conformational dynamics were likely the driving force behind differences in the NMR-derived networks. Indeed, our previous NMR studies indicated that the β2α2 and β6α6 loops were conformationally dynamic on multiple timescales (Axe and Boehr, 2013), but the millisecond timescale motions were suppressed in the *working* state (Axe and Boehr, 2013). In contrast, the picosecond-to-nanosecond timescale dynamics were rather ligand-independent (Axe and Boehr, 2013). Unfortunately, these previous studies were limited to Ala resonances, so could not provide information about the whole αTS enzyme.

To gain more insight into the relationships between structural dynamics and amino acid interaction networks in αTS, we characterized the millisecond timescale structural dynamics in the *resting, working*, indole-bound and G3P-bound states, using NMR ^15^N R_2_ relaxation dispersion experiments (Loria et al., 1999a,b). Interactions with ligands quenched some of the *resting* state motions, but induced motion in other areas of the enzyme. Many of the previously identified network residues displayed millisecond conformational exchange or were in close association with dynamic residues. This finding suggests that network changes can be relayed in part through changes in conformational motions.

## Results and discussion

### Conformational exchange events across the alpha subunit of tryptophan synthase

**Figure 2:**
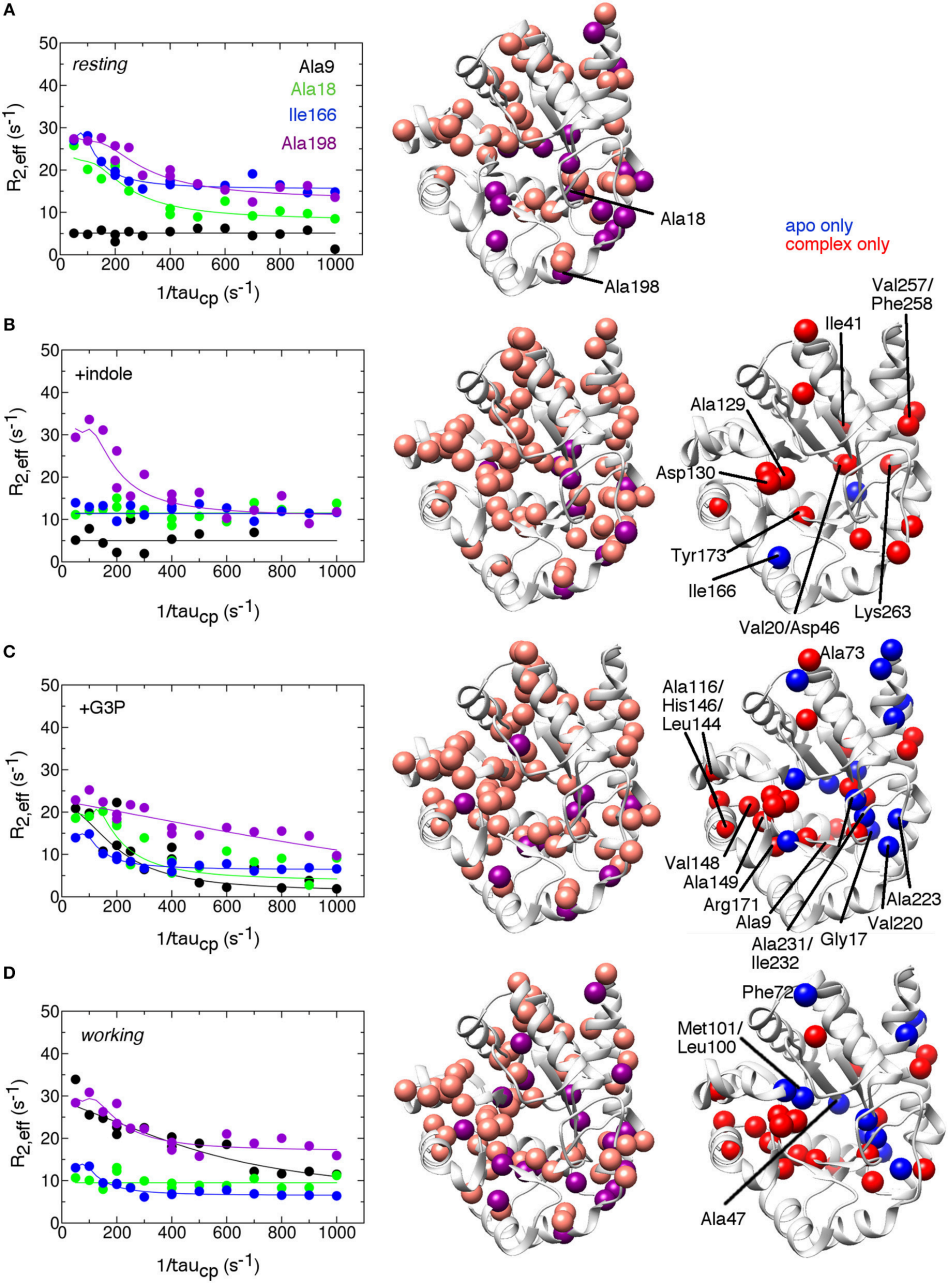
Conformational exchange events in the *E.coli* αTS enzyme **(A)** in the apo *resting* state, **(B)** bound to product indole, **(C)** bound to the product glyceraldehyde-3-phosphate (G3P) and **(D)** in the *working* state under catalytic turnover. The *working* state represents a 4:1 ratio of enzyme bound with substrate indole-3-glycerol phosphate (IGP) to enzyme bound with products indole and G3P (Axe and Boehr, 2013; Axe et al., 2014). (left) example ^15^N R_2_ relaxation dispersion curves collected at a ^1^H Larmor frequency of 850 MHz for the resonances belonging to Ala9 (black), Ala18 (green), Ile166 (blue), and Ala198 (purple). (middle) locations of conformational exchange according to the R_2_ relaxation dispersion experiments plotted as spheres onto the αTS structure. Here, we used the *S.typhimurium* αTS structure bound to glyceraldehyde-3-phosphate (PDB 2CLK) as it contains resolved β2α2 and β6α6 loops. Purple spheres indicate that associated R_2_ relaxation dispersion curves can be fit to two-site exchange, while pink spheres indicate exchange broadening, but the R_2_ relaxation dispersion curves cannot be fit reliably to two-site exchange. (right) a comparison of the conformational exchange events in the *resting* apo state compared to when αTS is bound with ligands. Blue (red) spheres indicate conformational exchange events present in the apo (ligand-bound) state but not in the ligand-bound (apo) state. Most of the amino acid residues associated with a change in conformational dynamics make contact and/or near each other in three-dimensional space. The R_2_ relaxation dispersion experiments were conducted at 283 K using a buffer consisting of 50 mM potassium phosphate, pH 7.8, 2 mM DTT, 0.2 mM Na_2_EDTA, and 10% ^2^H_2_O, and 0.5–1.0 mM protein with 10 mM indole and/or 20 mM G3P where appropriate.

To gain insight into conformational motions on the microsecond-to-millisecond timescale, we conducted ^15^N R_2_ relaxation dispersion experiments on αTS in various states, including the apo *resting* state, bound to the product indole (E:indole), bound to the product glyceraldehyde-3-phosphate (E:G3P), and under active turnover conditions in the *working* state (Figure 2). Active turnover conditions have been previously used to study conformational motions in the enzymes cyclophilin and adenylate kinase (Eisenmesser et al., 2002, 2005; Wolf-Watz et al., 2004).

We identified residues that were associated with more typical R_2_ relaxation dispersion curves that could be fit assuming two-site exchange (purple spheres in Figure 2), and other residues whose resonances displayed exchange broadening and/or whose R_2_ relaxation dispersion curves could not be reliably fit to kinetic or thermodynamic parameters (pink spheres in Figure 2). Some of the residues were also associated with higher than average ${\text{R}}_{2}^{0}$ values, which may be indicative of motions on a faster μs timescale. These experiments revealed that there were conformational exchange events throughout αTS under all conditions assessed, including in areas around the active site and in more distant regions, including on the outer α-helices. The pervasiveness of conformational exchange events around αTS would seem to be in contrast to previous X-ray crystal structure analyses of the *S. typhimurium* αTS-βTS, which indicated that ligand binding led to only local structural changes in αTS (Dierkers et al., 2009; Axe et al., 2014). A survey of β-factors in the X-ray crystal structures of isolated *E. coli* αTS (e.g., PDB 1XC4) also indicated that there was little dynamic disorder in the central β-strands, and the locations of conformational exchange identified by the ^15^N R_2_ relaxation dispersion experiments did not correspond to areas with higher β-factors. The conformational exchange events identified by the NMR experiments may be restricted in the crystal and/or reporting on a different timescale than the local atomic motions related to β-factors. The ^15^N R_2_ relaxation dispersion results on αTS are also in contrast to previous NMR relaxation studies on TIM itself, which indicated conformational exchange processes were generally localized around the β6α6 loop (Massi et al., 2006; Kempf et al., 2007).

### Dynamic networks in the alpha subunit of tryptophan synthase are switched on and off by the addition of ligands

The ^15^N R_2_ relaxation dispersion experiments on the apo *resting* state enzyme indicated that αTS is intrinsically dynamic on the μs-ms timescale (Figure 2). To gain more insight into how ligand interactions may modulate protein motions, we identified conformational exchange events that were only present in the *resting* state (blue spheres in Figure 2) and those that were only present when the enzyme was bound with the products or in the *working* state (red spheres in Figure 2). As a caveat to the following analysis, we note that the inability to detect conformational exchange by the ^15^N R_2_ relaxation dispersion experiments does not rule out conformational motions, but if present, these motions are outside the kinetic and thermodynamic windows accessible by these experiments. With this in mind, the presence of indole led to additional conformational exchange events around the indole-binding region (e.g., Ala129, Asp130 on the β4α4 loop, Tyr173 on the α5β6 loop), or otherwise near the active site (e.g., Val20 on the β1 strand). Indole binding also induced conformational exchange to more distant residues. Some of these residues are close to one another and can form potential linkages to residues at the active site. For example, Asp46 in the α1β2 loop is nearby Val20, but also nearby Ile41 in the α1 helix and Lys263 in the α8' helix, which is on the same α-helix as Val257 and Phe258. We do not expect that these indole-induced conformational exchange events are due simply to indole binding/release from the enzyme as we expect the enzyme to be fully saturated with indole under these conditions, and some of the indole-induced changes are similar to the changes induced by the presence of G3P, which we would not expect if the conformational exchange events were simply reporting on binding/release of ligands. However, we note that indole is released first in the αTS kinetic mechanism (Weischet and Kirschner, 1976), so the indole-bound complex may have limited functional relevance.

The binding of G3P both induces and represses conformational exchange events compared to the *resting* state enzyme (Figure 2). For example, the dynamic residue cluster associated with the indole-binding site encompassed additional residues, including Ala116 in the α3 helix, Leu144 in the α4 helix, His146 and Val148 in the α4β5 loop, Ala149 in the β5 strand, and Arg171 in the α5β6 loop. These dynamic connections may help to explain why G3P binding leads to large chemical shift changes in some of these associated resonances despite these amino acid residues being more distant from the G3P-binding site (Axe and Boehr, 2013). The presence of G3P also led to the loss of conformational exchange events around the active site and outlying regions. For example, Ile232 near the phosphate-binding site and nearby residues (e.g., Gly17 in the α0β1 loop, Val220, and Ala223 in the α7 helix, and Ala231 in the β8 strand) did not show conformational exchange in the presence of G3P. Binding of G3P may act to stabilize this region and suppress conformational dynamics. Overall, there appears to be distinct regions in αTS in which conformational motions are essentially switched “on” or “off” by the presence of G3P.

The *working* state induced very similar changes to the conformational exchange events as G3P binding (Figure 2). Nonetheless, conformational exchange events in the *working* state did not simply reflect a combination of the conformational exchange events in the indole-bound and G3P-bound complexes. Specifically, some of the dynamic clusters discussed above for the product-bound complexes were repressed or modified in the *working* state. Residues near the indole ring (i.e., Leu100, Met101, Ser125) and a cluster of amino acid residues in the α0β1 loop (Gly17), β1 strand (Ala18, Val20), α1β2 loop (Asp46, Ala47), α7 helix (Ala223), and β8 strand (Ala231 and Ile232) did not show conformational exchange in the *working* state. Residues in the extended β2α2 (Phe72, Ala73) and β6α6 (Leu191) loops also did not show conformational exchange, suggesting that these loops become less conformationally dynamic during catalytic turnover. The suppression of conformational dynamics near the indole-binding pocket and at the active site loops may be important for binding ligands and maintaining an environment conducive to chemical catalysis. With that said, it is interesting that Glu49 and other residues in the β2 strand remain conformationally dynamic. X-ray crystal structures suggest that there is a reorientation of Glu49 when IGP binds (Rhee et al., 1998); conformational exchange events in this region may be reporting in part on this reorientation.

**Figure 3:**
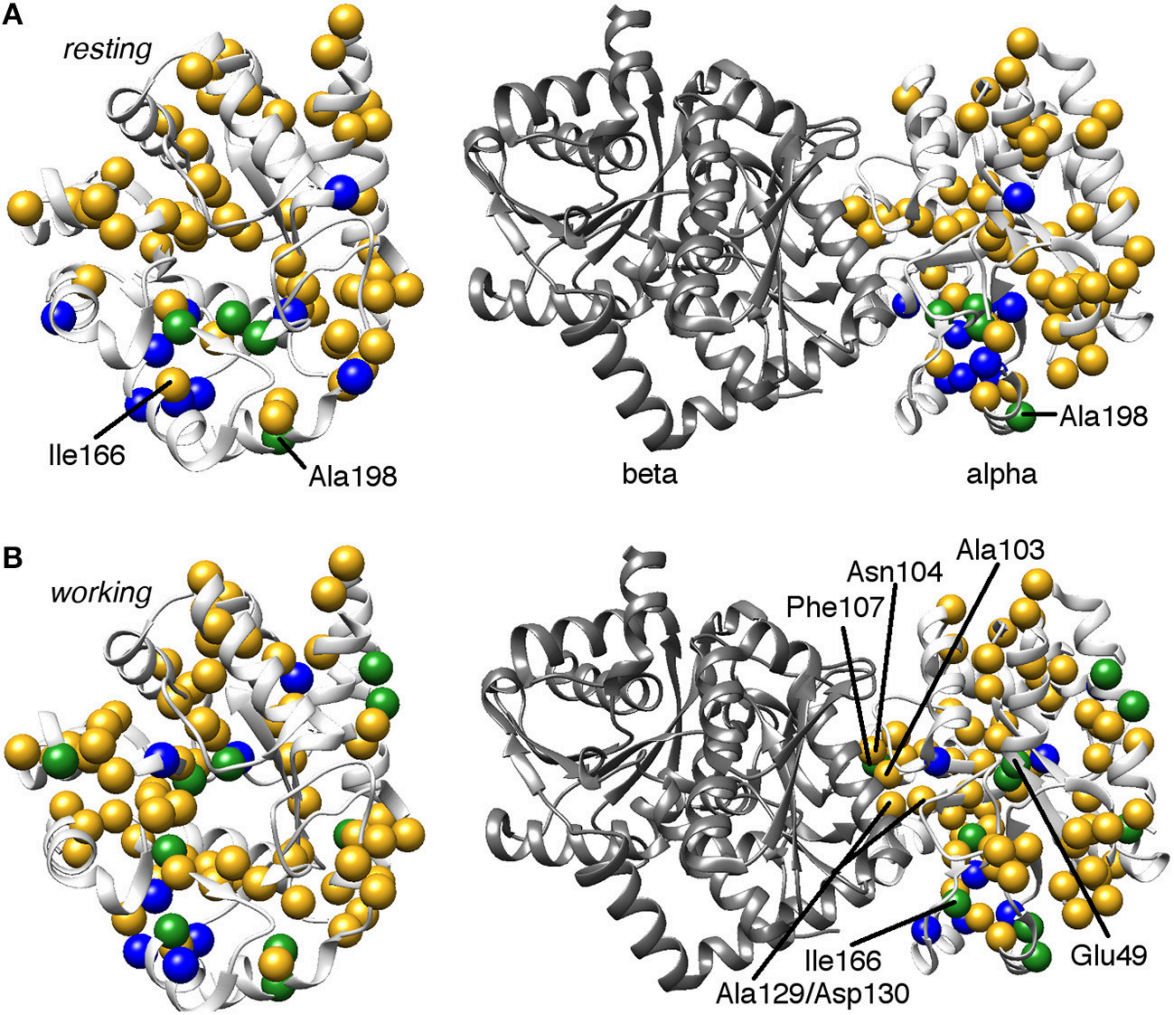
Comparison of conformational exchange events identified by ^15^N R_2_ relaxation dispersion experiments and a residue cluster identified by chemical shift covariance analysis (CHESCA) for the **(A)** apo *resting* and **(B)** *working* states of *E. coli* αTS. The *working* state represents active catalytic turnover conditions in which there is a 4:1 ratio of enzyme bound with substrate indole-3-glycerol phosphate (IGP) to enzyme bound with products indole and G3P (Axe and Boehr, 2013; Axe et al., 2014). Yellow spheres indicate μs-ms timescale conformational exchange events identified by ^15^N R_2_ relaxation dispersion experiments (i.e., pink and purple spheres in Figure 2), blue spheres indicate residues belonging to the previously identified CHESCA cluster, and green spheres indicate CHESCA cluster residues that show conformational exchange. The same information is presented on (left) the αTS structure and (right) the full TS complex (PDB 2CLK). It should be noted that all NMR data were collected in the absence of βTS. Nonetheless, there are conformational exchange events and CHESCA cluster residues near the βTS-binding interface, which may be important in the context of the full TS complex. There are more CHESCA cluster residues showing conformational exchange in the *working* state compared to the *resting* state.

It is also worth noting that residues at or near the αTS/βTS interface region, including the α3 helix and the β4α4 and β5α5 loops, showed millisecond conformational motions in all or most complexes (Figure 3). These regions may be sampling conformations conducive to interactions with βTS, or may simply be more flexible without the structural constraints imposed by βTS binding.

### Kinetic and thermodynamic assessment of the conformational exchange events in the alpha subunit of tryptophan synthase

In favorable circumstances, R_2_ relaxation dispersion experiments can also provide kinetics and thermodynamics information about the conformational exchange processes (Loria et al., 1999a,b). For two-site exchange between conformations A and B, R_2_ relaxation dispersion experiments can yield the exchange rate constant k_ex_ that is the sum of the forward (k_AB_) and reverse (k_BA_) rate constants, the populations of the exchanging conformations (p_A_, p_B_), and the dynamic chemical shift difference between the exchanging conformations (Δω = δω_A_-δω_B_). Unfortunately, many of the ^15^N R_2_ relaxation dispersion curves would not fit according to a two-site exchange model and/or one or more data points were sufficiently off the fitted curve, likely owing to noise, to have confidence in the fitted parameters. Those residues that could fit reliably to a two-site exchange model had similar exchange kinetics and populations, so these residues were fit with global k_ex_ and p_A_/p_B_ values (Table 1) and residue-specific Δω values. The *resting*, indole-bound and *working* states all had similar global k_ex_ values, whereas the G3P-bound state had a substantially lower global k_ex_ value. It should be noted that the rate constants reported here (e.g., k_A_ for the *working* state is 15 s^−1^ at 283 K) are substantially larger than the k_cat_ for αTS (k_cat_ ~ 0.01 s^−1^ at 298 K). In other enzymes, such as dihydrofolate reductase (Boehr et al., 2006) and ribonuclease A (Beach et al., 2005), the dynamic chemical shift differences (i.e., Δω values) were used to indicate that these enzymes fluctuate into previously identified states (Boehr et al., 2009), however this was not the case for αTS (data not shown).

**Table 1:** Kinetic and thermodynamics parameters for the millisecond motions in the alpha subunit of tryptophan synthase at 283 K.

**State**	**k_ex_ (s^−1^)**	**p_A_ (%)**	**p_B_ (%)**	**k_AB_ (s^−1^)**	**k_BA_ (s^−1^)**
*Resting*	286 ± 58	94.2	5.8	17	269
+indole	287 ± 25	90.0	10.0	29	254
+G3P	146 ± 21	93.0	7.0	10	136
*Working*	273 ± 19	94.4	5.6	15	258

### Comparison of chemical shift-based amino acid interaction networks and conformational exchange events in the alpha subunit of tryptophan synthase

We had previously identified amino acid interaction networks in αTS using the CHESCA (chemical shift covariance analysis) method (Axe et al., 2014). In short, we identified clusters of amino acid residues that responded in a similar fashion to a set of Ala-to-Gly perturbations, those being A59G, A67G, A158G, A180G, and A185G where Ala59 and Ala67 are on the extended β2α2 loop, Ala158 is on the β5α5 loop, and Ala180, and Ala185 are on the β6α6 loop. The β2α2, β5α5, and β6α6 loops are all known to contribute to αTS-βTS communication and indole channeling (Lim et al., 1991a,b; Hiraga and Yutani, 1997; Dunn, 2012). Intriguingly, the composition of one of these clusters substantially changed between the *resting* and *working* states (Axe et al., 2014). We proposed that this cluster was especially important for αTS function, considering that the catalytic residue Glu49 becomes part of this cluster only in the *working* state. Molecular dynamics (MD) simulations also indicated that Glu49 has coordinated motional trajectories with this *working* state cluster (Ai et al., 2010; Fatmi and Chang, 2010). With this in mind, we focused only on the CHESCA cluster that changes between the *resting* and *working* states of αTS.

**Figure 4:**
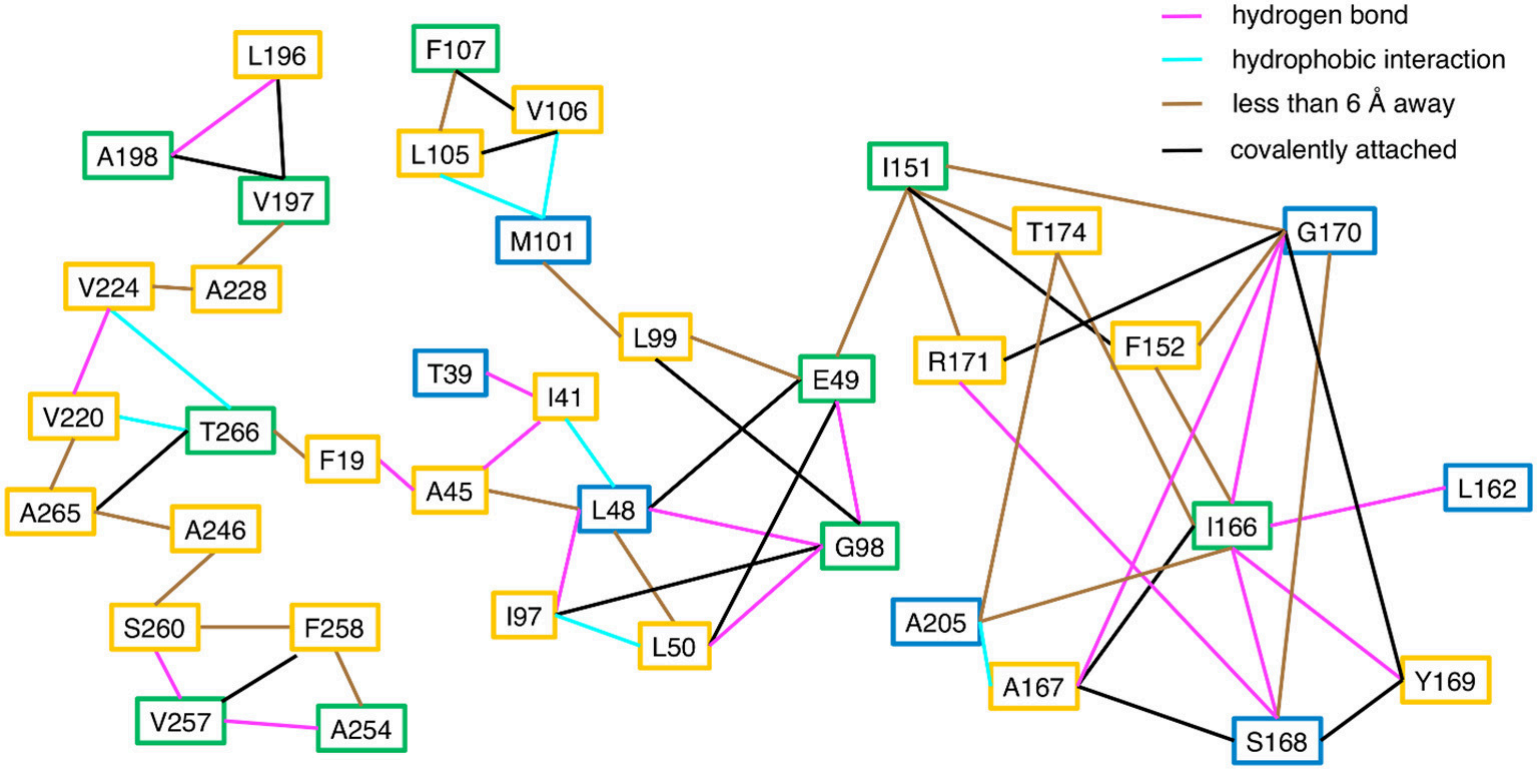
Connections between CHESCA network and exchanging residues in the *working* state enzyme. Residues that show millisecond conformational exchange according to ^15^N R_2_ relaxation dispersion studies are boxed in yellow, residues that were previously identified to be CHESCA network residues (Axe et al., 2014) are boxed in blue, and residues that are CHESCA network residues and show conformational exchange are boxed in green. Lines indicate the type of interaction between the residues, including hydrogen bond (pink line), a hydrophobic interaction (cyan line), covalently attached (black line), or within at least 6 Å distance where side chain motion may lead to contact. CHESCA network residues either undergo conformational exchange or interact with residues that undergo conformational exchange.

Consistent with the MD simulations, only a few CHESCA cluster residues showed conformational exchange according to the ^15^N R_2_ relaxation dispersion experiments (Figure 3, green spheres), whereas the majority of the CHESCA cluster residues showed conformational exchange in the *working* state. In fact, the CHESCA cluster residues that did not show conformational exchange (Figure 3, blue spheres) in the *working* state were immediately adjacent to other residues that were conformationally dynamic on the μs-ms timescale (Figure 3, yellow spheres). Especially in the *working* state, paths of interactions can be traced connecting the CHESCA cluster residues through residues undergoing conformational exchange (Figure 4).

## Conclusion

We had previously identified amino acid interaction networks in the isolated αTS subunit, in the absence of the βTS subunit. These networks were different between the *resting* and *working* states of the enzyme. Unfortunately, the changes in the networks could not be explained by structural considerations alone considering the structural similarity between ligand-free and ligand-bound αTS (at least in the context of the αTS-βTS complex) (Axe et al., 2014). However, MD simulations suggested that network residues had coupled motions (Axe et al., 2014), prompting the study of protein structural dynamics by NMR. Indeed, the ^15^N R_2_ relaxation dispersion studies indicated that the *resting* and *working* states of the enzyme had some different dynamic features on the μs-ms timescale. Ligand binding appeared to enable or suppress the millisecond motions of paths or clusters of residues, or in the very least, changed which residues have detectable conformational exchange within the limits of the ^15^N R_2_ relaxation dispersion experiments. Previously identified network residues were conformationally dynamic, or interacted with dynamic residues, suggesting that information through these networks may be conducted by changes in conformational motions in the absence of detectable structural changes. These results suggest networks in proteins (at least those identified by CHESCA) do not simply reflect structural interactions in the lowest energy, ground-state conformation, but rather these networks also reflect motional coupling and/or interactions as proteins fluctuate into higher energy protein conformations. This network view is more complex than the “mechanical linkage” model (Yu and Koshland, 2001) in which allosteric signals propagate through sequential structural changes in a “domino-like” fashion, and is more compatible with dynamically driven allostery (Cooper and Dryden, 1984; Reinhart et al., 1989; Petit et al., 2009; Motlagh et al., 2014; Kornev and Taylor, 2015; Nussinov and Tsai, 2015; Tzeng and Kalodimos, 2015; Guo and Zhou, 2016; Saavedra et al., 2018).

The dynamic networks identified in isolated αTS may also be important in the context of the full αTS-βTS complex. Some motions were induced near the αTS/βTS binding interface upon binding ligands (e.g., Ala129, Asp130), whereas other residues were structurally dynamic also in the apo state (e.g., Asn104). Amino acid substitutions at these positions are known to affect αTS-βTS function and stability (Lim et al., 1991a,b; Yang et al., 2007). Computer simulations also suggested that interactions between αTS and βTS in the full complex are dependent on ligand binding (Fatmi and Chang, 2010), which might be reflected by a change in conformational dynamics. The T183V substitution, known to affect substrate channeling, also affects the CHESCA networks (Axe et al., 2015), and amino acid substitutions at network positions affect αTS function (Axe et al., 2014). Analysis of network substitutions may also reveal how the αTS networks are important for function in the full αTS-βTS complex. However, it should be kept in mind that binding of βTS may change or restrict these motions, which would likely have further dynamic effects throughout αTS. A comparison of αTS structural dynamics in the absence and presence of βTS may reveal those motions most important for communication within the full enzyme complex. Unfortunately, we were unable to collect ^15^N R_2_ relaxation dispersion data for the full αTS-βTS complex, likely owing to its large size (i.e. 143 kDa). Nonetheless, preliminary studies suggest that ^13^C-methyl experiments may be possible for the full enzyme complex similar to studies in other large protein complexes (Velyvis et al., 2009a,b). These studies may reveal dynamic networks in αTS-βTS similar to the dynamic networks revealed in another substrate-channeling enzyme imidazole glycerol phosphate synthase (ImGPS), involved in histidine biosynthesis. In ImGPS, allosteric pathways were initially identified based on community analysis of MD simulations (Rivalta et al., 2012). Amino acid substitutions at network positions in HisH resulted in the suppression of millisecond timescale dynamics and decreased enhancement of HisF catalytic activity (Lisi et al., 2017). These studies also suggest that network changes can be conducted through changes in conformational exchange dynamics in the absence of notable ground-state structural differences.

## Materials and methods

### Overexpression and purification of αTS samples

All samples were overexpressed using *Escherichia coli* BL21(DE3^*^) cells grown in either Luria-Bertani media (EMD Millipore, Billerica, MA, USA) or M9 minimal media for NMR experiments. Samples for backbone relaxation dispersion were grown in ^2^H_2_O-based media with ^15^N-labeled ammonium chloride (Cambridge Isotopes, Tewksbury, MA, USA).

All samples were purified using an anion-exchange column Q-sepharose (GE Healthcare, Pittsburgh, PA, USA) with buffers A (25 mM HEPES, pH 7.5, 1 mM Na_2_EDTA) and B (buffer A with 1 M NaCl) using a 0–50% gradient of buffer B. Samples were concentrated with a Corning Spin-X UF centrifugal concentrator (Sigma Aldrich, St. Louis, MO, USA) to a volume of ~1 mL then purified on a S100 gel filtration column (GE Healthcare) in buffer A with 200 mM NaCl.

### NMR sample preparation and experiments

Following purification, a ZEBA desalting column (Thermo Fisher) was used to exchange all ^15^N-labeled samples into NMR buffer (50 mM potassium phosphate, pH 7.8, 2 mM DTT, 0.2 mM Na_2_EDTA, and 10% ^2^H_2_O). The samples contained 0.5–1.0 mM protein with 10 mM indole (Thermo Fisher), 20 mM G3P (Sigma Aldrich), and/or 3 mM IGP where appropriate. All experiments were collected on Brüker (Billerica, MA, USA) Avance III spectrometers.

### ^15^N R_2_ relaxation dispersion experiments

^15^N R_2_ relaxation dispersion experiments were collected at 283K on 600 and 850 MHz spectrometers. R_2_ relaxation rates were measured using relaxation-compensated CPMG (Carr-Purcell-Meiboom-Gill) pulse sequences in a constant time manner (Loria et al., 1999a,b). The total CPMG period was 40 ms. Where appropriate, data was fit to the following equations for exchange between two sites:

$$\begin{array}{l} R_{2}\left(\frac{1}{\tau_{CP}}\right)=R_{2}^{0}+\frac{1}{2}[k_{ex}-\frac{1}{\tau_{CP}}{cosh}^{-1}(D_{+}cosh\left(\eta_{+}\right)\\ -D_{-}cos\left(\eta_{-}\right))] \end{array}$$

Where,

$$\begin{array}{l} D_{\pm }=\frac{1}{2}{\left[\pm 1+\frac{\Psi +2\Delta \omega^{2}}{{\left(\Psi^{2}+\varsigma^{2}\right)}^{1/2}}\right]}^{1/2}\;and{\;\eta }_{\pm }\\ \;=\frac{\tau_{CP}}{2}{\left[\pm \Psi +{\left(\Psi^{2}+\varsigma^{2}\right)}^{1/2}\right]}^{1/2} \end{array}$$

using the computer program GLOVE (Sugase et al., 2013). In these equations, Ψ = *kex*^2^ – Δω^2^, ζ = −2 Δωk_ex_(p_A_–p_B_), τ_CP_ is the time between 180° pulses in the CPMG segment, $R_{2}^{0}$ is the *R*_2_ relaxation rate in the absence of conformational exchange, p_A_ and p_B_ are the populations of states A and B, *k*_ex_ is the rate of exchange between states A and B, and Δω is the difference in the chemical shift between states A and B.

## Data availability

The raw data supporting the conclusions of this manuscript will be made available by the authors, without undue reservation, to any qualified researcher.

## Author contributions

KO, JA, and DB contributed to the conception and design of the study. KO, JA, and RD generated protein samples. KO and JA collected ^15^N R_2_ relaxation dispersion data. DS supplied technical advice with the implementation of the ^15^N R_2_ relaxation dispersion experiments. KO and DB generated figures. KO and DB wrote the manuscript. All authors contributed to manuscript revision, read and approved the submitted version.

### Conflict of interest statement

The authors declare that the research was conducted in the absence of any commercial or financial relationships that could be construed as a potential conflict of interest.
